# Effect of pH-Shift Treatment on IgE-Binding Capacity and Conformational Structures of Peanut Protein

**DOI:** 10.3390/foods13213467

**Published:** 2024-10-29

**Authors:** Qin Geng, Wenlong Zhou, Ying Zhang, Zhihua Wu, Hongbing Chen

**Affiliations:** 1 State Key Laboratory of Food Science and Resources, Nanchang University, Nanchang 330047, China; 2 College of Food Science and Technology, Nanchang University, Nanchang 330047, China; 3 Sino-German Joint Research Institute, Nanchang University, Nanchang 330047, China

**Keywords:** peanut protein, pH-shift treatment, structure change, IgE-binding capacity

## Abstract

Hypoallergenic processing is an area worthy of continued exploration. In the treatment of the peanut protein (PP), pH shift was applied by acidic (pH 1.0–4.0) and alkaline (pH 9.0–12.0) treatment, after which the pH was adjusted to 7.0. Following pH-shift treatment, PP showed a larger particle size than in neutral solutions. SDS-PAGE, CD analysis, intrinsic fluorescence, UV spectra, and surface hydrophobicity indicated the protein conformation was unfolded with the exposure of more buried hydrophobic residues. Additionally, the IgE-binding capacity of PP decreased after pH-shift treatment on both sides. Label-free LC–MS/MS results demonstrated that the pH-shift treatment induced the structural changes on allergens, which altered the abundance of peptides after tryptic digestion. Less linear IgE-binding epitopes were detected in PP with pH-shift treatment. Our results suggested the pH-shift treatment is a promising alternative approach in the peanut hypoallergenic processing. This study also provides a theoretical basis for the development of hypoallergenic food processing.

## 1. Introduction

Peanut allergy is a type of food allergy reaction that affects many individuals. Peanuts belong to the legume family, which is known for its high nutritional value and contains more protein than other nuts [1]. The mature seeds of peanuts are rich in lipids and proteins, with a ratio of 49% and 25%, respectively. Nonetheless, peanut proteins are a source of various highly allergenic proteins, and they comprise 17 separate families of allergens (Ara h 1–18; Ara h 4 is proven to be a subunit of Ara h 3) [2]. Peanut allergy is a prototypical immunoglobulin E (IgE)-mediated immune response caused by the ingestion of peanut allergens, resulting in allergic reactions within the gastrointestinal tract or throughout the body [3]. Even minimal exposure to peanuts can elicit severe allergic responses. At present, peanuts are extensively utilized as primary ingredients or additives in popular food products and various packaged foods such as peanut butter, peanut milk, candies, and pastries [4]. Avoiding the dietary intake of foods containing peanut-derived components poses remarkable challenges for individuals susceptible to peanut allergens. The prevalence and incidence of peanut allergies increased annually, thereby posing a substantial risk to food safety for affected individuals [5].

Numerous studies have been conducted to develop hypoallergenic peanut-based foods using diverse food processing techniques such as heat, enzyme, ultrasonic, and pressure treatment [6,7,8,9]. However, only a few methods meet commercial feasibility criteria concerning nutrition content, flavor profile preservation, cost-effectiveness, and safety considerations. The allergenicity of allergens strongly relied on their epitopes, which depended on their 3D structure and secondary structure [8]. Thus, disrupting these structures can not only reduce potential sensitization but also minimize impacts on nutritional value and taste attributes of food products.

Recently, numerous studies have examined the conformational changes in dietary protein molecules via unfolding a protein structure in an extreme acidic or alkaline environment and refolding in the following neutral environment, a phenomenon known as pH-shift treatment [10,11,12]. This technique was considered an economical and environmentally friendly method because it can eliminate the need for dangerous chemical solvents and minimize energy consumption [13]. Under extreme pH conditions (pH 2.0 and 12.0), soy oligomeric globulin structure at quaternary level tends to unfold and dissociate into subunits. Subsequently, the dissociated subunits may further unfold, forming a novel flexible polypeptide that is intermediate between the native and denatured states when the pH of the protein suspensions is readjusted to neutral [11]. The low immunoreactivity of black turtle bean (*Phaseolus vulgaris* L.) protein isolate was observed after low pH-shift treatment (pH 1.0–3.0) [14]. In addition, recent studies reported that pH shift (pH 1.0–3.5) prior to autoclaving (121 °C, 15 min) was a beneficial treatment for red kidney bean lectin with lower antigenicity and good digestibility [15]. To our best knowledge, the effect of pH-shift treatment on the peanut protein (PP) was not investigated. Perhaps pH-shift treatment is a promising technique for mediating sensitization of peanut protein.

Thus, in order to study the effect of pH-shift treatment on IgE-binding capacity and conformational structures of peanut protein (PP), the PP was treated by pH-shift treatment (pH 1.0–12.0). The structural change in PP was evaluated by sodium dodecyl sulfate-polyacrylamide gel electrophoresis (SDS-PAGE), intrinsic fluorescence, extrinsic fluorescence, ultraviolet (UV) absorption spectroscopy, and circular dichroism (CD). Various protein and peptide distributions were studied by label-free LC–MS/MS. The structural alteration of the allergen was investigated by molecular dynamics simulation. In addition, the potential antigenicity and digestion ability were further evaluated by enzyme-linked immunosorbent assay (ELISA) and in vitro digestibility. This study would provide valuable contributions to the hypoallergenic food processing of peanuts.

## 2. Materials and Methods

### 2.1. Materials

Peanuts were bought from the local market (Jiangxi, Nanchang, China). 1-Anilinonaphthalene-8-sulfonic acid (ANS) was acquired from Sigma-Aldrich Chemical Inc. (St. Louis, MO, USA). Trypsin was obtained from Promega Inc. (Madison, Wisconsin, USA). Phosphate buffer (PB) was obtained from Solarbio (Beijing, China). High-purity methanol, acetonitrile, and formic acid (FA) for mass spectrum analysis were obtained from Thermo Fisher Scientific (China) Co., Ltd. (Shanghai, China). Deionized water was used to make the solutions, and all other compounds used were analytical grade.

### 2.2. pH-Shift Treatment of PP

Peanut protein was obtained in accordance with our previous study [16]. PP was treated at room temperature using the pH-shift method based on the study of Li, Wu, and Wang [17] with some modifications. In brief, 1 g of PP powder was dissolved into 100 mL of distilled water to obtain the solution. Afterward, the pH of the solution was adjusted to 1.0, 2.5, 4.0, 9.0, 10.5, and 12.0 using 1 mol/L HCl or 1 mol/L NaOH for a 4 h incubation at room temperature. Then, the treated PPs were adjusted to 7.0 by using 1 mol/L HCl or 1 mol/L NaOH. Then, the dispersion was dialyzed to remove the Na^+^ and Cl^−^ and lyophilized for 48 h. The treated PP were marked as pH_1.0_-shift, pH_2.5_-shift, pH_4.0_-shift, pH_9.0_-shift, pH_10.5_-shift, and pH_12.0_-shift. Neutral PP, as the control, was marked as NPP. The treated PPs were diluted to an adequate concentration for the following measures.

### 2.3. Structural Characterization

#### 2.3.1. Sulfate-Polyacrylamide Gel Electrophoresis (SDS-PAGE)

Electrophoresis was performed in accordance with a previously described method at 1 mg/mL PP samples [18]. After electrophoresis, the gel was stained with Coomassie Brilliant Blue for 1 h, and the destained gel was visualized and photographed using a GS-800 densitometer (Bio-rad Laboratories, Inc., Hercules, CA, USA).

#### 2.3.2. Particle Size and Zeta Potential

The particle size and zeta potential of PP samples were measured using a Zetasizer Nano instrument (Malvern Instruments Ltd., Malvern, UK) [19]. The control and pH-shift-treated PP samples were diluted with distilled water to 0.1 mg/mL.

#### 2.3.3. Circular Dichroism (CD)

The CD spectroscopy PP samples were characterized using a CD spectrometer (MOS-450, Claix, France) in the far-UV range (190–250 nm), in accordance with the method of Li, Zhao, and Li [12]. The secondary structure changes in PP samples were evaluated at a scanning speed of 50 nm/min with a path length of 1.0 mm. The concentration of the samples was set at 0.1 mg/mL. The online CONTIN tool in DichroWeb was used to calculate the content of the secondary structure.

#### 2.3.4. Fluorescence Spectroscopy

The intrinsic fluorescence spectrum of the samples was measured using a fluorescence spectrophotometer (F-7000, Hitachi, Tokyo, Japan). The excitation wavelength was set at 280 nm with a slit width of 2.5 nm, and the emission spectrum ranging from 290 to 500 nm with a slit width of 2.5 nm was collected [20]. An appropriate protein concentration of PP solutions at 0.5 mg/mL was selected to mitigate inner filter effects during the fluorescence experiment.

#### 2.3.5. UV Spectroscopy

UV absorbance spectra of PP samples were measured using a spectrophotometer (UV-2450, Shimadzu, Kyoto, Japan) ranging from 200 to 400 nm equipped with a 1.0 cm path length cell, following the method outlined by Teng, Zhang, and Dai [21]. The absorption spectrum for a PB blank was subtracted from that of the sample.

#### 2.3.6. Surface Hydrophobicity

The ANS was used as the probe of fluorescence to investigate the change in surface hydrophobicity. The ANS probe binding of PP was measured in accordance with a previous report with some modifications [22]. A total of 5 mL of PP sample was mixed with 100 μL of 8 mM ANS solution. The mixture was kept in the dark for 15 min. The quartz cuvette with a light path of 1 cm was adopted. The excitation wavelength was set as 390 nm, and the emission wavelength ranging from 400 to 600 nm was collected.

### 2.4. ELISA

The IgE-binding activity of the PP samples was assessed using indirect competitive ELISA as described in a previous study [23]. In brief, 100 μL 10 μg/mL NPP sample was added to individual wells of a 96-well plate and incubated overnight at 4 °C. Then, the wells were washed five times with PBS tween-20 (PBST) for 5 min each time. Subsequently, a blocking buffer (250 μL/well) was added and incubated at 37 °C for 2 h. Then, PBST washes were performed. Next, a pH-shift-treated PP sample (0.5–10 μg/mL) and 50 μL of serum extracted from patients allergic to peanuts (diluted at a ratio of 1:300) were added to each well (100 μL), followed by incubation at 37 °C for 1 h. Then, the wells were washed again with PBST five times before adding an HRP-conjugated goat anti-human IgE antibody (100 μL, diluted at a ratio of 1:20,000). This step involved incubating the plates at 37 °C for 1 h. After washing the wells as previously described, a TMB (3, 3′, 5, 5′-Tetramethylbenzidine) developer solution (100 μL per well) was added under incubation at 37 °C for 15 min. Finally, a 2 M H_2_SO_4_ solution (50 μL) was added to stop the enzyme-catalyzed reaction. The absorbance at 450 nm was measured using a UV–visible spectrophotometer.

The following equation was used to analyze the IgE Inhibition Ratio:

$$Inhibition\;Ratio(\%)=(1-\left(\frac{B_{1}-B_{0}}{B_{2}-B_{0}}\right))\times 100$$

Here, *B*_0_ refers to the absorbance values of blank wells; *B*_1_ represents the absorbance value of the sample wells to be tested; *B*_2_ represents the absorbance values of positive wells.

### 2.5. LC–MS/MS

Label-free peptide samples were prepared and digested as described in the study of Rost et al. [24] with slight modifications. Proteins from each sample separated and visualized on the SDS-PAGE gel were further sliced and diced into fractions. The stained gel pieces were destained with 200 µL of 50% acetonitrile/50 mmol/L NH_4_HCO_3_ and dehydrated with 100% acetonitrile reduced with 150 µL of 10 mM DTT at 56 °C for 60 min. Subsequently, the protein fractions were alkylated with 150 µL 100 mM iodoacetamide for 45 min in the dark (room temperature). Subsequently, the protein fractions were tryptic digestion with trypsin (100 ng/µL sequencing grade) incubated for 30 min at 4 °C, then topped up with 20 µL 25 mM NH_4_HCO_3_. After digestion, peptides from each protein fraction were extracted by adding 100 µL of 30% acetonitrile/0.1% for 15 min. The peptide digests were lyophilized and reconstituted into 20 µL of 0.1% formic acid, vortexed, and then centrifuged prior to mass spectrometry. A total of 4 μL of the supernatant was aspirated and identified by LC–MS. The samples were desalted by using a Zip Tip C_18_ column (Millipore Inc., Bedford, MA, USA). The peptide mixture was separated using a PepMap RSLC C_18_ column (Thermo Fisher Scientific, Waltham, MA, USA) under buffer A (0.1% formic acid) to buffer B (0.1% formic acid in acetonitrile) at 350 nL/min and then identified using a Q Exactive Plus-Orbitrap MS (Thermo Fisher Scientific, Waltham, MA, USA). Finally, the collected dates were searched in the database using PEAKS software (PEAKS Studio 11).

### 2.6. Molecular Dynamics Simulation

In accordance with the Uniprot ID of allergen, the 3D structure file (.PDB) of the allergen monomer after Alphafold simulation was downloaded from the Uniprot website as the initial PDB file. The protein structure at different pH values can be simulated using the web of H^++^ [25]. Molecular dynamics simulation was performed using GROMACS 2019 software (Stockholm Center for Biomembrane Research, Stockholm, Sweden) according to previous research [16]. Proteins with pH values of 1.0, 7.0, and 12.0 were utilized as the initial file for the GROMACS simulation. During molecular dynamics simulation, CHARMM36 force field was applied, and the TIP3P water model was used for solvation. Then, molecular dynamics simulations run for 10 ns. The final structure PDB file was imported into PyMOL 2.5.5 software to visualize.

### 2.7. Digestibility Analysis

Digestibility analysis was performed according to previous research [26]. A 1 mL, 1 mg/mL PP sample was mixed with 0.4 mL of simulated gastric fluid (SGF) solution and incubated for 80 min at 37 °C with a pepsin-to-substrate protein (10 U:1 μg). The digestion reactions were terminated at pH 7.5 by adding 0.2 M Na_2_CO_3_. At certain intervals of 0, 10, 20, 40, and 80 min, a 20 μL PP sample was collected for Tricine–SDS-PAGE analysis. During simulated intestinal digestion, the simulated intestinal fluid (SIF) solution was added to the PP samples digested in SGF for 80 min with a gastric digestive production ratio of 3:1 (*v*/*v*). After incubation for 40 min at 37 °C, the digestion was stopped by heating at 100 °C for 5 min. A total of 20 μL of sample was collected at 0, 5, 10, 20, and 40 min for Tricine–SDS-PAGE analysis. The IgE-binding activity of the samples digested simulation after SIF was assessed by ELISA as described in Section 2.4.

### 2.8. Statistical Analysis

For the results, mean values ± standard deviations were used. IBM SPSS (version 27.0, IBM Inc., Armonk, New York State, USA) was used to conduct statistical analysis. Significant differences between means (*p* < 0.05) were examined using a one-way ANOVA and the Duncan test.

## 3. Results and Discussion

### 3.1. Structural Characterization

#### 3.1.1. SDS-PAGE

**Figure 1:**
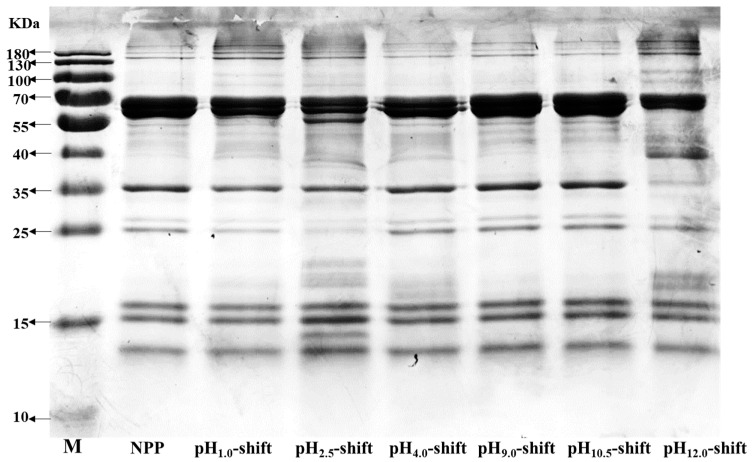
SDS-PAGE of PP after different pH-shift treatments.

Figure 1 shows the subunit information of PPs. Several characteristic bands of major allergens for PP can be distinctly identified on SDS-PAGE, including Ara h 1 (64 kDa), Ara h 2 (17–20 kDa), Ara h 3 (43/38/36/24 kDa), and Ara h 6 (15 kDa) [18]. Compared with the neutral PP (NPP), no significant change in the electrophoretic band of PP was observed at pH_4.0_-shift, pH_9.0_-shift, and pH_10.5_-shift PP. However, the electrophoretic bands changed greatly after extreme acidic (pH 1.0, pH 2.5) and extreme alkaline (pH 12.0) cycles. After pH 2.5 shift, the protein bands about 64 and 25 kDa were significantly weakened, and two new bands about 38 and 15 kDa appeared. These results indicated that peanut protein could be degraded under pH 2.5 shift conditions [27]. This result was consistent with that of Li, Wu, and Wang [17]. Peanut protein was denatured intensely under pH 2.0. The visible increase in the protein bands around 38 and 20 kDa for the pH_12.0_-shift sample indicated that PP hydrolysis or protein composition alterations occurred for extreme pH treatment [28]. Wang, Wen, and Wang [28] found that extreme pH treatment can induce a series of structural changes for soy protein. Meanwhile, pH_1.0_-shift, pH_2.5_-shift, and pH_12.0_-shift proteins displayed distinct dark bands at the top of the separating gel, indicating the existence of protein aggregates. This result was similar to that of Li, Zhu, and Wu [25], which indicated adjusting pH to neutral can aggregate the extended molecular chains in the protein.

#### 3.1.2. Particle Size and Zeta Potential

**Figure 2:**
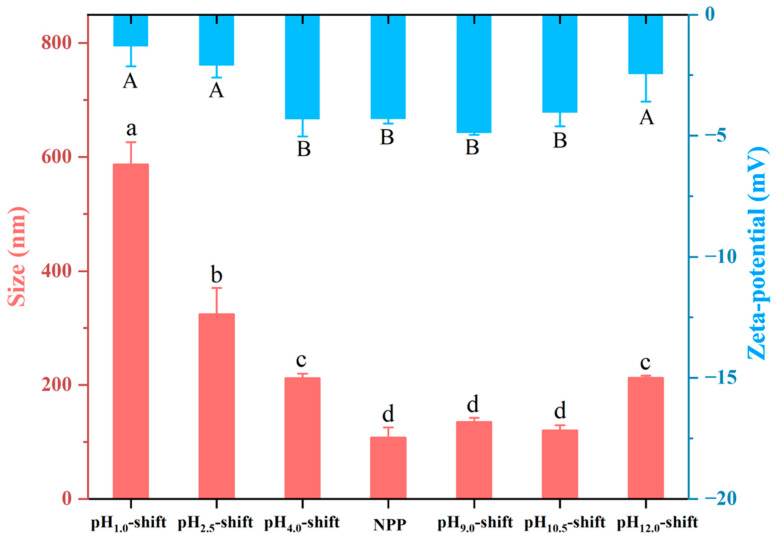
Particle size and zeta potential of PP after different pH-shift treatments. “A”, “B”, and “a–d” indicated the values of zeta potential and size had a significant difference (*p* < 0.05) between different samples.

Figure 2 showed the diameter of the PP sample, which was treated by different pH shifts. Compared with the NPP (107.92 nm), PP treated by pH shifting had a larger size. The mean diameter of PP particles in acidic treatment was increased from 212.10 to 587.45 nm and in alkaline treatment from 135.05 to 212.40 nm. The finding suggested that the pH treatment had a potential effect on the unfolding of peanut protein. The result was consistent with the research of Wang, Wen, and Wang [28], who reported that larger particles of soy protein after acid–alkali treatment were observed. As shown in Figure 2, the zeta potential of the NPP and PP treated by different pH-shift conditions was negative. However, the NPP sample had the highest zeta potential, probably because the PP in its natural state had the lowest energy and was most stable. Furthermore, when the pH (like pH = 1.0 or pH = 12.0) used is extreme, the absolute value of zeta potential is low. The particle size and zeta potential values were consistent with research on the characterization of a pH-treated soy protein [28]. The extreme acidic pH caused the protein structure to unfold, and then the unfolded PP aggregated via hydrophobic interactions. Thus, the aggregates were larger and with less charge density than NPP. While at pH 12.0, SDS-PAGE indicated decomposition of PP, and small fragments cannot form big aggregates. In this case, the increased particle size of PP may be due to the moderate expansion of the structure rather than forming aggregates.

#### 3.1.3. Fluorescence Spectroscopy

**Figure 3:**
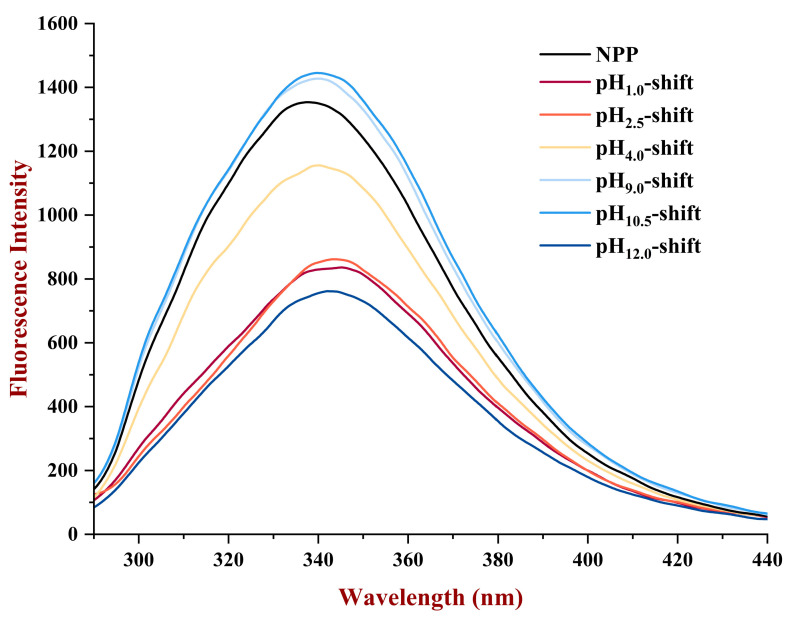
The emission fluorescence spectra of PP after various pH shifting.

The changes in fluorescence intensity can be used to study the changes in the tertiary structure of proteins after pH treatments. The emission fluorescence spectra of proteins correspond to the presence of tryptophan (Trp) and tyrosine (Tyr) residues. The emission spectra of PP were shown in Figure 3. The maximum fluorescence intensity of PP with a single peak was obtained near 340 nm. Compared with the maximum emission wavelength of NPP at 338 nm, a blue shift of the maximum emission wavelength and lower fluorescence intensity were observed at 342–345 nm for the pH_1.0_-shift, pH_2.5_-shift, and pH_12.0_-shift treatment. Meanwhile, the fluorescence intensity of the PP reduced after acidic shift and pH_12.0_-shift treatment, which could be explained by protein unfolding after extreme pH treatment, thereby inducing a microenvironmental change of protein. This result was consistent with the result of particle size.

#### 3.1.4. UV

**Figure 4:**
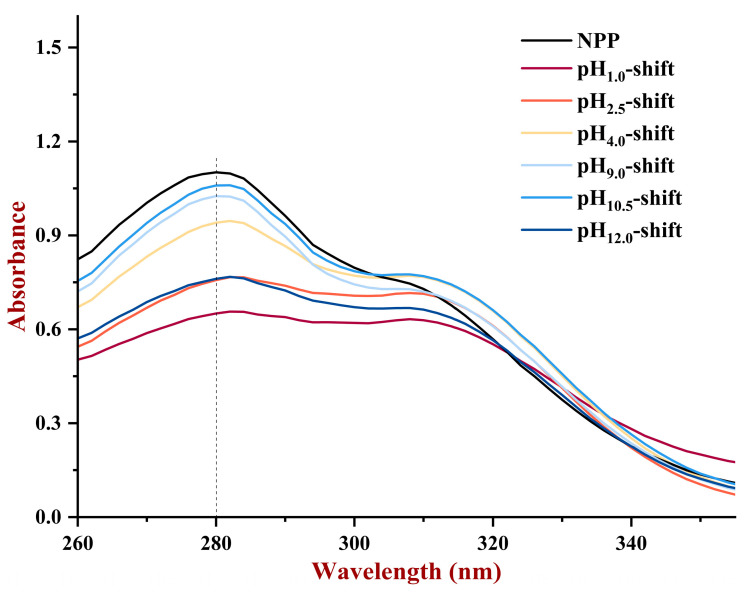
The UV spectra of PP after various pH shifting.

UV spectroscopy is a common method for examining changes in the protein microenvironment. As shown in Figure 4, the UV spectra of the PP treated by a pH shift had peaks at 280 nm, which were caused by the tryptophan and tyrosine chromophores of proteins. As shown in Figure 4, after pH-shift treatment, the absorption intensity of PP samples at 280 nm was reduced, especially the sample after pH_1.0_-shift, pH_2.5_-shift, and pH_12.0_-shift treatment, indicating the microenvironmental change in PP was induced by extreme pH treatment and resulted in the burial of UV-absorbing group [28]. The UV spectroscopy results were consistent with those of fluorescence analysis, indicating the formation of reaggregated structures when PP was unfolded under extreme acid or alkaline treatment.

#### 3.1.5. CD

**Figure 5:**
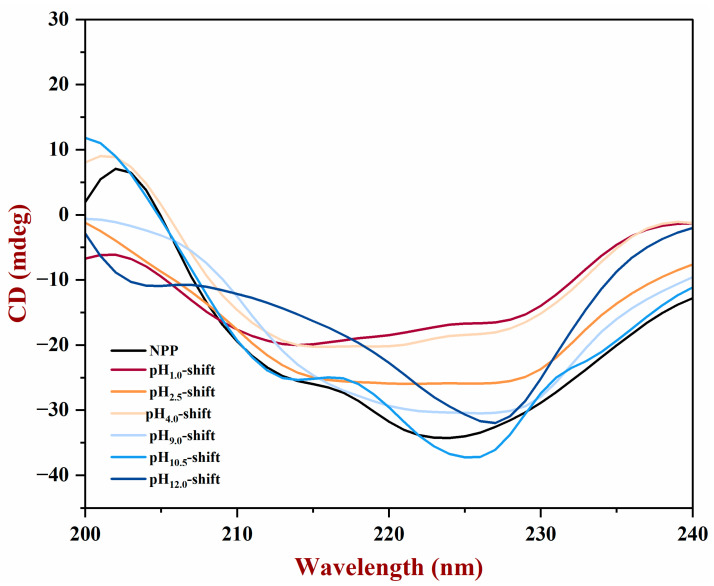
CD spectra of PP after different pH-shift treatments.

The secondary structures of peanut protein were measured using the circular dichroism. Figure 5 showed that the NPP had a negative peak at 210–230 nm. The spectra intensity values of acidic pH-shift-treated PP were higher than those of NPP, while the spectral features of alkaline pH-shift-treated PP were obviously changed to the NPP. This phenomenon may be due to the modifications to the molecular microenvironment of the protein, which led to changes in hydrophilicity or hydrophobicity and the displacement of the chromophore absorption peak [29]. The different proportions of the secondary structures of the samples were shown in Table 1. The result indicated a mild decline in α-helix content and an increase in β-sheet after pH-shift treatment, which suggested an increase in the structural flexibility of proteins [15]. Furthermore, considering that the α-helix structure was a structural characteristic of epitopes, some of the epitopes would be destroyed when the α-helix of PP decreased [2]. Consequently, the loss of α-helix would lead to the breakdown and elimination of epitopes, which could reduce the allergic reaction.

#### 3.1.6. Surface Hydrophobicity

**Figure 6:**
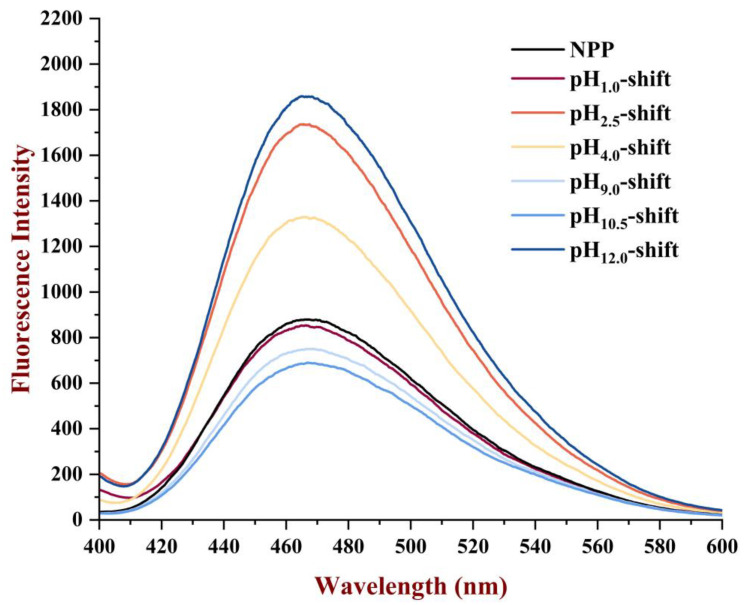
Surface hydrophobicity (H_0_) of PP after different pH-shift treatments.

As shown in Figure 6, the surface hydrophobicity (H_0_) was measured to the change in hydrophobic groups touched on the protein surface. The result showed that H_0_ of PP treated by extreme acidic and extreme alkaline pH (pH 2.5-, 4.0-, and 12.0-shift treatment) evidently increased compared with the NPP. Presumably, the surface of PP was rearranged after the pH-shift treatment, and the more hydrophobic region of PP was exposed. The polar environment of the treated PP induced the conformational recombination, and hydrophobic amino acid residues were exposed as a result of the protein’s intermolecular hydrogen bonds being broken and electrostatic repulsion being increased [25]. Similar results were obtained for red kidney bean lectin under acidic pH-shift treatment with an increase in surface hydrophobicity for the expansion and refolding of hydrophobic regions when the red kidney bean lectin was treated by extreme acidic treatment [15].

### 3.2. ELISA

**Figure 7:**
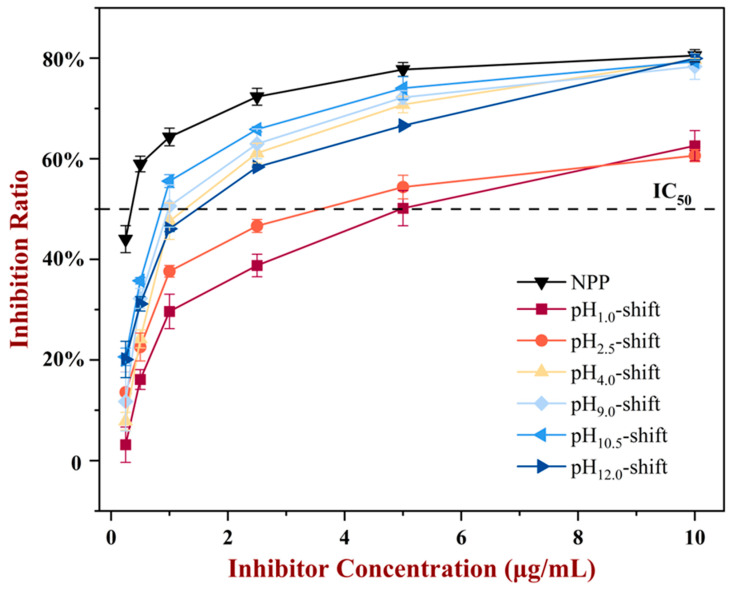
The IgE binding capacity of PP after different pH-shift treatments.

In the indirect competition ELISA experiment, the IC_50_ value represents the strength of antigen–antibody binding ability. A lower IC_50_ value of the competing antigen indicated a higher binding capacity of the antigen to the antibody since less of the antigen was needed to reach the half-inhibitory ratio. The results shown in Figure 7 indicated that the NPP showed the strongest positive antigenicity with the lowest IC_50_ (0.22 ± 0.04 μg/mL), and the IgE-binding ability of the pH-shift-treated PP decreased with higher IC_50_ (pH_1.0_-shift: 4.55 ± 0.03 μg/mL, pH_2.5_-shift: 3.86 ± 0.18 μg/mL, pH_4.0_-shift: 1.42 ± 0.22 μg/mL, pH_9.0_-shift: 1.18 ± 0.06 μg/mL, pH_10.5_-shift: 1.10 ± 0.15 μg/mL, pH_12.0_-shift: 1.66 ± 0.12 μg/mL). Moreover, a remarkable decrease in IgE-binding ability was observed with the increase in alkaline pH value and the decrease in acidic pH value, which could be explained by the conformational changes. It was clear that the pH_1.0_-shift and pH_2.5_-shift produced PP aggregators, thus lowering the binding efficiency. However, the pH_4.0_-shift, pH_9.0_-shift, and pH_10.5_-shift samples had not caused serious denaturation, and then PP showed a close result to NPP. At pH 12.0, PP was degraded, and small fragments or peptides showed the reduction in antigenicity.

### 3.3. Protein and Peptide Profiles

To evaluate the changes in the peptide profile of PP after pH-shift treatment, label-free LC–MS/MS was conducted to analyze the protein and peptide profiles. The peptide data collected by LC–MS/MS were matched with the peptide fingerprint of the Uniprot database (Table S1), and the major peanut allergens were successfully matched in samples: Ara h 1 (P43238), Ara h 2 (Q6PSU2), Ara h 3 (O82580), and Ara h 6 (Q647G9). Table 2 showed the abundance of four main allergens of peanut (Ara h 1, 2, 3, 6) about pH_1.0_-shift and pH_12.0_-shift treatments.

**Figure 8:**
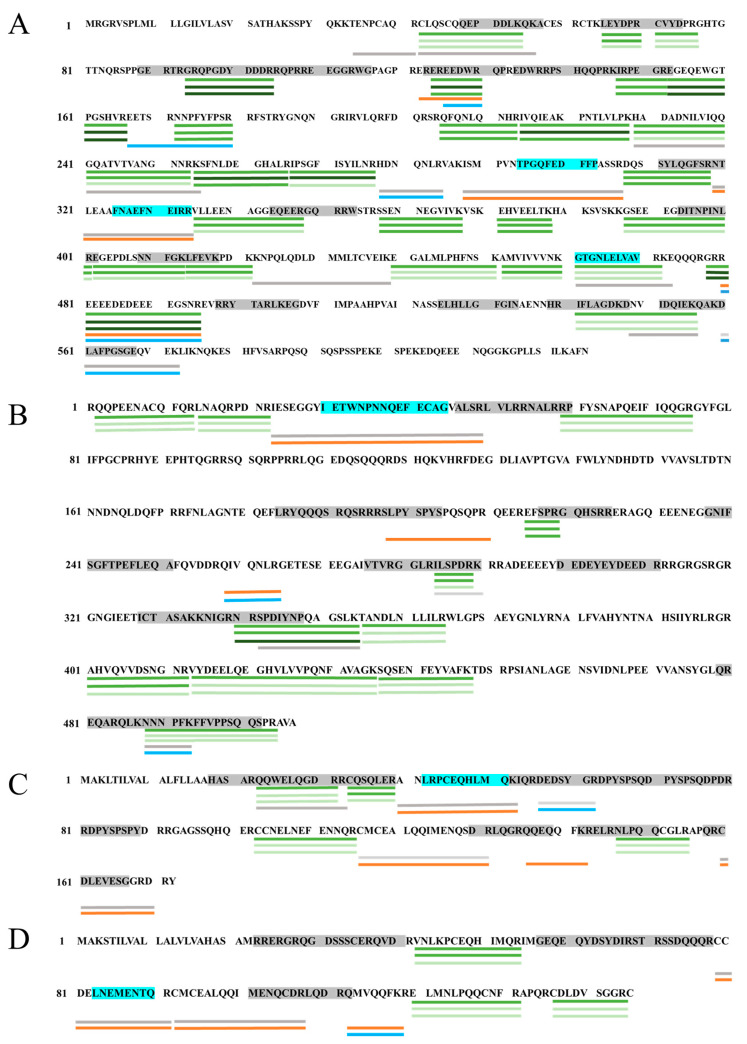
Changes in peptide maps of four main peanut allergens after pH_1.0_-shift and pH_12.0_-shift treatment. (**A**): Ara h 1; (**B**): Ara h 3; (**C**): Ara h 2; and (**D**): Ara h 6. The line under the amino acid sequence represented the peptides that were detected and matched to the allergen. Green line: peptides were detected in all three samples; from top to bottom were NPP, pH_1.0_-shift PP, and pH_12.0_-shift PP. The relative abundance of peptides from pH-shift-treated PP to NPP was defined as RA. Green: 0.5 < RA < 2, no significant change in abundance; dark green: RA > 2, there was a significant increase in abundance; RA < 0.5, there was a significant decrease in abundance. Gray line: the peptide was only detected in NPP; orange line: the peptide was only detected in pH_1.0_-shift PP; blue line: the peptide was only detected in pH_12.0_-shift PP. The linear IgE epitopes were shown in shadow. The linear IgE epitopes in blue represented the epitopes that were detected intact.

Figure 8 shows the peptide coverage and abundance of the four main allergens, the change of which reflected the restriction site exposed or covered, as well as the linear epitopes. The protein sequences of allergens were searched by Uniprot, and the linear epitopes were matched with the reports [30,31,32,33]. Peptides abundant from NPP were considered at a level of 1, and the peptides with a relative abundance of >2.0 or <0.5 in treated peanuts were considered significantly increased or decreased [34].

As shown in Table S1 and Figure 8, a total of 47 peptides were detected in Ara h 1, and the peptide coverage was about 60%. After pH-shift treatment with acid and alkali, the abundance of the detected peptides in PP was lower than that in NPP, which may be due to the change in trypsin restriction site caused by pH shifts. In addition, the peptide length might not be under the detection threshold because of protein cross-linking or breaking (the peptide detection length is 5–45 amino acids). Some peptides, such as 94–104 (with a relative abundance of 11.5 in Ara h 1 of pH_1.0_-shift PP and 3.8 in Ara h 1 of pH_12.0_-shift PP), showed an increase in abundance following pH-shift treatment, while some peptides, such as 122–130, were present only in the treated protein. This finding could be due to the fact that the restriction sites, such as R^93^ and R^121^, were exposed, making protein peptides easier to digest in the pH-shift-treated PP. On the contrary, the relative abundance of some peptides reduced remarkably or disappeared in pH_1.0_-shift PP such as 278–284 and 560–572, as well as in pH_12.0_-shift PP peptides such as 288–307, compared with Ara h 1 in NPP. This result may be due to the restriction site masked in PP after treatment, including R^277^ and K^559^ in pH_1.0_-shift PP and K^287^ in pH_12.0_-shift PP. Three linear IgE-binding epitopes were found intact in Ara h 1 of NPP: 294-TPGQFEDFFP-303(1), 325-FNAEFNEIRR-334(2), and 461-GTGNLELVAV-470(3) [30]. Epitopes 1, 2, and 3 were reduced in pH_12.0_-shift PP, and epitopes 1 and 3 were reduced in pH_1.0_-shift PP, which may explain the IgE-binding capacity of PP decreased treated by different pH shifts.

The peptide abundance of Ara h 3 in NPP accounted for 40%. After pH shifting by acid and alkali, the abundance of Ara h 3 peptides was reduced. Compared with Ara h 3 in NPP, the restriction sites R^196^ and R^257^ were exposed in pH_1.0_-shift, as well as R^257^ and R^339^ in pH_12.0_-shift. Some peptides were not detected, which indicated that the restriction sites were masked after a pH shift, such as K^290^, R^341^, and K^487^ in pH_1.0_-shift, and R^22^, K^290^, and R^341^ in pH_12.0_-shift. Linear IgE-binding epitope ^29^IETWNPNNQEFECAG^44^ was involved in the detected peptides [32].

The peptide coverage of Ara h 2 was about 49%. Based on the abundance of peptides of Ara h 2 in samples, the restriction sites R^32^ and R^55^ were masked, and R^135^ was exposed in pH_1.0_-shift. However, the peptide coverage of pH_12.0_-shift PP was lower than that of NPP, and the abundance of detected peptides decreased to 34%. This finding may be due to the fact that the restriction sites R^32^, R^39^, R^115^, and R^59^ were masked in pH_12.0_-shift PP. Compared to NPP, the decreased peptides involved the linear IgE-binding epitope ^42^LRPCEQHLMQ^51^ [33].

The peptide coverage of Ara h 6 was about 43%. The peptide coverage increased to 58% in the pH_1.0_-shift sample. However, it decreased to 28% when the pH shifted to a pH of 12.0. Compared to NPP, peptides 112–118 were only detected in treated proteins, which indicated that the restriction site R^111^ was exposed to Ara h 6 after a pH shift. Less peptides were detected in pH_12.0_-shift PP, and the restriction sites R^78^ and R^91^ were masked in Ara h 6. Moreover, the decreased peptides included linear epitopes ^83^LNEMENTQ^90^ [31].

Therefore, these results demonstrated that pH-shift-induced structural changes on the allergens, thereby altering the cleavage site of trypsin and the peptide abundance detected by LC/MS–MS. This result was consistent with the SDS-PAGE and structural change results. Compared to NPP, less linear IgE-binding epitopes were detected in pH-shift PP, which may cause the sensitization changes in PP.

### 3.4. Molecular Dynamics Simulation

**Figure 9:**
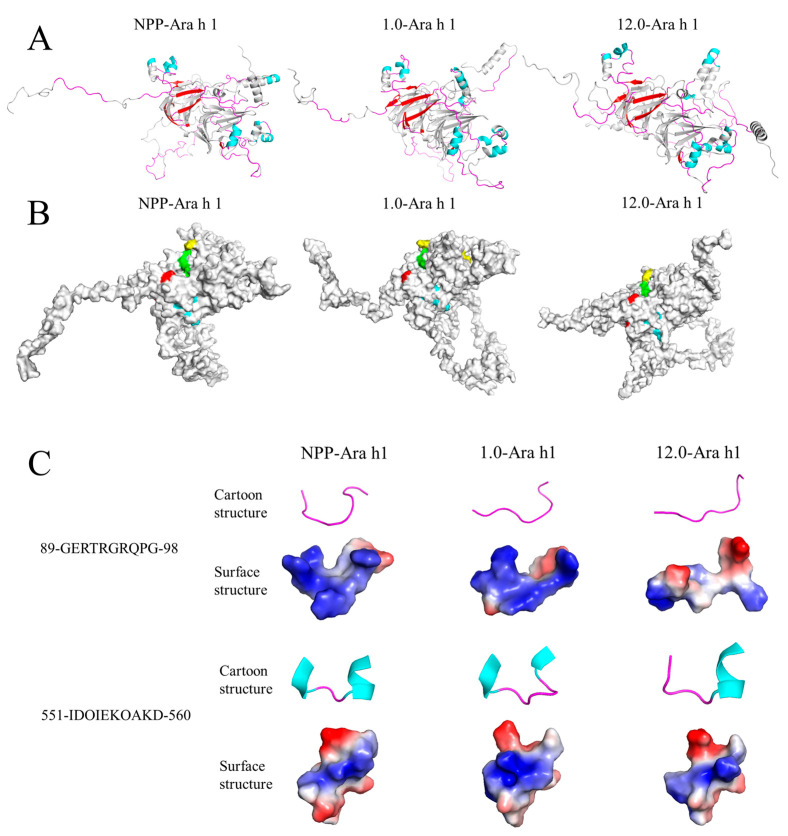
The secondary and tertiary structure changes in Ara h 1 after a pH shift. (**A**): The simulated cartoon structure with secondary structure and linear IgE epitopes of Ara h 1 in NPP, pH_1.0_-shift PP (1.0), pH_12.0_-shift PP (12.0). The linear IgE epitopes in colors represent different secondary structures: cyan: helix; red: sheet; magenta: loop. (**B**): The simulated surface structure with comformational IgE epitopes of Ara h 1 in NPP, pH_1.0_-shift PP (1.0), pH_12.0_-shift PP (12.0). (**C**): The secondary structure and the electrostatic potential distribution of portion linear IgE-binding epitopes in Ara h 1, in surface structure, blue: positive potential and red: negative potential.

In this study, a molecular dynamics simulation of Ara h 1 was performed to demonstrate the structural alteration of the allergen in a graphical manner. Figure 9 AB illustrates the secondary and tertiary structural changes in Ara h 1 after pH_1.0_-shifting (1.0-Ara h 1) and pH_12.0_-shifting (12.0-Ara h 1). The secondary structure of Ara h 1 changed after a pH shift with a mild decline in α-helix (such as amino acids 551–560) content and an increase in β-sheet (such as amino acids 578–586). The tertiary structure of Ara h 1 had loosened. These results were consistent with the structural characterization. Figure 9 C shows the secondary structure and electrostatic potential distribution of a portion of linear IgE-binding epitopes (89-GERTRGRQPG-98 and 551-IDOIEKOAKD-560). Compared with NPP-Ara h 1, the secondary structure of epitopes 89–98 changed slightly, all of which were loop structures, while the positive potential distribution on the surface of 12.0-Ara h 1 changed to neutral. This change in structure may correspond to the exposure of the abovementioned restriction site R^93^. For epitope 551–560, the restriction site K^559^ had an α-helix structure in NPP-Ara h 1 and 12.0-Ara h 1, but this α-helix structure loosens in 1.0-Ara h 1. The change in secondary and tertiary structures may explain the peptide fingerprint results of the restriction site K^559^ masked in 1.0-Ara h 1 but not in 12.0-Ara h 1. These results indicated that pH shifts induced differences in secondary and tertiary structures on the epitopes.

### 3.5. Digestibility Analysis

**Figure 10:**
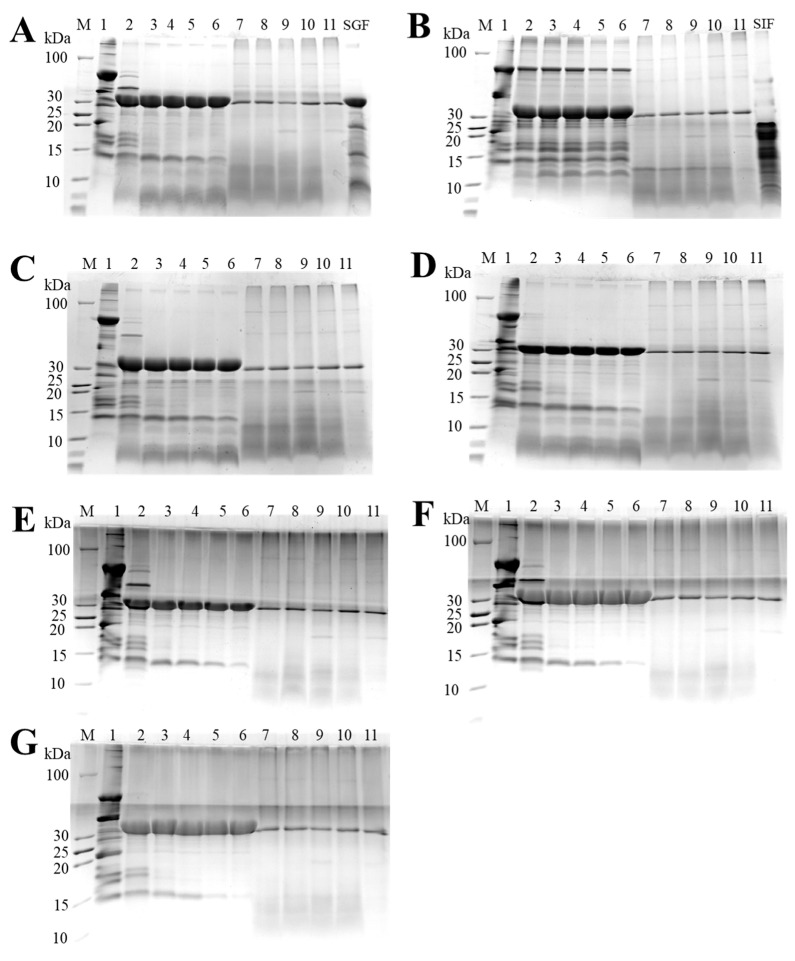
The in vitro digestion of NPP and pH-shift-treated PP. (**A**–**G**) were NPP, pH_1.0_-shift PP, pH_2.5_-shift PP, pH_4.0_-shift PP, pH_9.0_-shift PP, pH_10.5_-shift PP, and pH_12.0_-shift PP, respectively. Lane M, protein marker; Lane 1, PP; Lanes 2–6, simulated gastric digestion of 0, 10, 20, 40, and 80 min, respectively; Lanes 7–11, simulated intestinal digestion of 0, 5, 10, 20, and 40 min, respectively; Lanes SGF, simulated gastric fluid solution; and Lanes SIF, simulated intestinal fluid solution.

As immunoreactivity is related to protein integrity during digestion, the stability of digestion and the IgE-binding capacity of the digestion products were examined in this study. Figure 10 showed the results of in vitro digestibility. As shown in Figure 10A, the NPP band intensity decreased gradually as the duration of gastric digestion increased. The band intensity of Ara h 2 and Ara h 6 remained intact for a long time, particularly Ara h 6, indicating the strong resistance to pepsin digestion for disulfide bridges [35]. The digestion rate of PP after pH 2.5, 4.0, 9.0, and 10.5 shifting maintained a relatively consistent state with NPP during gastric digestion. However, pH_1.0_-shift PP was particularly resistant to proteolysis, as the band intensity of pH_1.0_-shift PP did not change remarkably during digestion, and the bands with a high molecular weight (>100 kDa) enhanced. This result may be due to PP aggregation after pH_1.0_-shift, which was consistent with the conformational changes in particle size, whereas pepsin restriction sites may reduce and epitopes, including conformational and linear, may be retained, which may affect the potential allergenicity of the PP [32]. On the contrary, the digestion rate of pH_12.0_-shift PP was markedly expedited. The bands of pH_12.0_-shift-treated PP almost disappeared after 10-min digestion by pepsin, except for Ara h 6. Combined with the results of SDS-PAGE, pH_12.0_-shift PP showed reaggregation and partial fragmentation, and more pepsin cleavage sites were exposed, which would destroy epitopes, thereby reducing sensitization.

**Figure 11:**
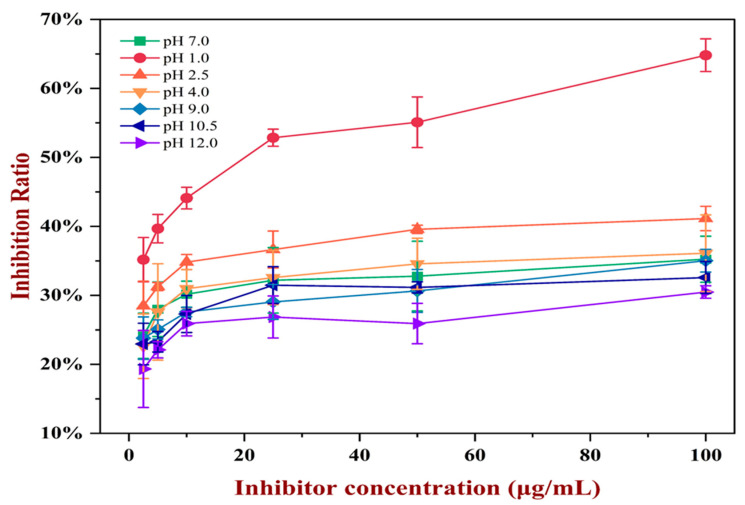
The IgE binding capacity after digestion of NPP and pH-shift-treated PP.

After digestion, the IgE-binding capacity of the products of gastrointestinal digestion was investigated (Figure 11). The IgE-binding ability of PP digestion products treated after acidic pH shifting increased with the decrease in acidic pH value. However, the IgE-binding ability of PP digestion products treated after alkaline pH shifting decreased with the increase in alkaline pH value. This result was consistent with the gastrointestinal digestion performance of PP. The digestion rate of pH_1.0_-shift and pH_2.5_-shift PP slowed down for aggregators, thus lowering the digestion efficiency, which may result in the retention of more epitopes, thereby increasing the IgE-binding ability [36]. Furthermore, following an alkaline pH shift, the digestion rate of PP increased, and epitopes may be destroyed, which decreased the IgE-binding capacity.

## 4. Conclusions

This study revealed that pH-shift treatment induced a structural change in peanut proteins, especially extreme pH-shift treatment, which noticeably affected its potential allergenicity. Upon the pH-shift treatment, the structure of PP unfolded with an increase in particle size, and hydrophobic amino acids were exposed in rearrangement. The IgE binding ability decreased for epitopes masked or destroyed. Alkali-treated PP may exhibit a faster rate of digestion and a lower IgE binding ability on the products of gastrointestinal digestion, whereas acid-treated PP may show a lower digestion rate and a higher IgE binding ability on products of gastrointestinal digestion. The pH-shift treatment is a promising alternative approach in the peanut hypoallergenic processing. The study on the relationship between protein structure and its potential sensitization provides a theoretical basis for the development of promising hypoallergenic food processing.

## Figures and Tables

**Table 1:** The secondary structure content of PP after different pH-shift treatments. Values with the same letters in the same line are not significantly different (*p* < 0.05).

Secondary Structure	NPP	pH_1.0_-Shift	pH_2.5_-Shift	pH_4.0_-Shift	pH_9.0_-Shift	pH_10.5_-Shift	pH_12.0_-Shift
α-helix	32.10% ± 2.26 a	26.95% ± 1.76 b	27.06% ± 1.29 b	25.10% ± 2.95 b	28.55% ± 2.19 b	29.76% ± 0.57 ab	25.9% ± 2.32 b
β-sheet	17.13% ± 2.40 b	21.83% ± 0.45 a	22.57% ± 2.31 a	22.8% ± 2.40 a	20.9% ± 2.36 a	18.76% ± 0.46 b	21.53% ± 2.36 a
β-turn	15.73% ± 0.41 c	18.33% ± 0.35 a	17.10% ± 0.17 b	16.37% ± 0.32 c	16.57% ± 0.25 c	16.43% ± 0.05 c	16.16% ± 0.61 c
Random coil	36.73% ± 0.61 a	31.23% ± 2.09 b	36.93% ± 2.29 a	35.66% ± 0.80 a	34.9% ± 0.55 a	35.03% ± 0.06 a	36.27% ± 1.41 a

**Table 2:** The amino acid coverage and peptide numbers of four main allergens in NPP, pH_1.0_-shift PP, and pH_12.0_-shift PP.

Allergen	Accession	Neutral PP		pH_1.0_-Shift PP		pH_12.0_-Shift PP	
		Coverage (%)	Peptides	Coverage (%)	Peptides	Coverage (%)	Peptides
Ara h 1	P43238	61	47	55	43	56	42
Ara h 2	Q6PSU2	49	9	49	9	34	6
Ara h 3	O82580	40	20	37	17	34	16
Ara h 6	Q647G9	43	5	58	7	28	4

## Data Availability

The original contributions presented in the study are included in the article/Supplementary Materials, further inquiries can be directed to the corresponding author.

## Supplementary Materials

The following supporting information can be downloaded at: https://www.mdpi.com/article/10.3390/foods13213467/s1. Table S1: List of peptides in four peanut allergens from NPP, pH_1.0_-shift PP, and pH_12.0_-shift PP detected by LC/MS–MS. Area: Peak areas of peptides.
