# Supplementary material for: Effect of pH-Shift Treatment on IgE-Binding Capacity and Conformational Structures of Peanut Protein
Source: Foods. 2024 Oct 29;13(21):3467. doi: 10.3390/foods13213467 (PMC11545115; doi:10.3390/foods13213467)
Supplement: Supplementary file 1 [file foods-13-03467-s001.zip › foods-3266159-supplementary.pdf]

Effect of pH-shift treatment on IgE-binding capacity and conformational structures of peanut protein

Qin Geng[1,2], Wenlong Zhou[1,2], Ying Zhang[1,2], Zhihua Wu[*1, 3], Hongbing Chen[1, 3]

1 State Key Laboratory of Food Science and Resources, Nanchang University, Nanchang, China

2 College of Food Science and Technology, Nanchang University, Nanchang, China

3 Sino-German Joint Research Institute, Nanchang University, Nanchang, China

Correspondence

Zhihua Wu, State Key Laboratory of Food Science and Resources, Nanchang University, 235 Nanjing Rd. East, Nanchang 330047, China.

Email: wuzhihua@ncu.edu.cn

Table S1: List of peptides in four peanut allergens from NPP, $pH_{1.0}$-shift PP and $pH_{12.0}$-shift PP detected by LC/MS–MS.

allergen	number	Peptide	Area	Area	Area
			**$pH_{1.0}$-shift PP**	**NPP**	**$pH_{12.0}$-shift PP**
**Ara h 1**	1	R.QPGDYDDDR.R	3.30E+05	8.97E+04	1.17E+05
	2	K.HADADNILVIQQGQATVTVANGNNR.K	4.84E+06	6.04E+06	3.24E+05
	3	R.DQSSYLQGFSR.N	1.55E+07	1.67E+07	1.32E+07
	4	R.GRQPGDYDDDRR.Q	2.34E+06	1.99E+05	7.49E+05
	5	R.QFQNLQNHR.I	2.54E+07	1.50E+07	1.48E+07
	6	R.EEEEDEDEEEEGSNR.E	1.44E+06	3.27E+06	1.95E+06
	7	R.QPGDYDDDRR.Q	1.92E+06	3.39E+05	3.64E+05
	8	R.EGEQEWGTPGSHVR.E	8.29E+06	4.55E+06	4.38E+06
	9	R.VLLEENAGGEQEER.G	2.54E+07	2.56E+07	1.37E+07
	10	R.IFLAGDKDNVIDQIEK.Q	1.30E+07	1.87E+07	2.03E+06
	11	R.EGEQEW(+15.99)GTPGSHVR.E	4.34E+05	2.79E+04	4.90E+05
	12	K.HADADNILVIQQGQATVTVANGNNRK.S	0	3.20E+05	
	13	K.EGALMLPHFNSK.A	8.13E+06	1.15E+07	4.48E+06
	14	R.IVQIEAKPNTLVLPK.H	2.11E+07	7.11E+06	6.19E+06
	15	K.SFNLDEGHALR.I	2.87E+07	3.82E+07	3.96E+07
	16	K.DLAFPGSGEQVEK.L	0	1.78E+07	1.12E+07
	17	K.ISMPVNTPGQFEDFFPASSR.D	9.91E+05	6.05E+06	
	18	R.KSFNLDEGHALR.I	2.59E+06	8.81E+05	1.18E+06
	19	R.IPSGFISYILNR.H	1.20E+06	5.83E+05	1.04E+05
	20	K.LFEVKPDKK.N	1.67E+07	1.34E+07	8.57E+06
	21	K.KGSEEEGDITNPINLR.E	8.41E+06	7.62E+06	3.57E+06
	22	R.NTLEAAFNAEFNEIRR.V	1.53E+06	1.23E+05	0
	23	R.EGEPDLSNNFGK.L	1.39E+07	1.45E+07	7.42E+06
	24	R.SSENNEGVIVK.V	8.37E+06	6.28E+06	4.14E+06
	25	K.GTGNLELVAVR.K	5.92E+07	7.10E+07	3.77E+07
	26	R.REEEEDEDEEEEGSNR.E	2.01E+06	0	1.31E+06
	27	K.AMVIVVVNK.G	9.96E+06	1.20E+07	5.48E+06
	28	R.GRREEEEDEDEEEEGSNR.E	2.68E+06	4.58E+05	1.17E+06
	29	R.GRQPGDYDDDR.R	3.74E+05	5.62E+04	1.77E+05
	30	R.EGEQEWGTPGSH(+15.99)VR.E	2.05E+05	2.02E+05	2.37E+05
	31	K.LEYDPR.C	1.68E+05	0	0
	32	K.EHVEELTK.H	8.63E+06	5.98E+06	3.99E+06
	33	K.LFEVKPDK.K	1.51E+07	1.69E+07	1.05E+07
	34	K.KNPQLQDLDMMLTC(+57.02)VEIK.E	0	5.52E+05	0
	35	R.NNPFYFPSR.R	1.65E+07	1.18E+07	1.28E+07
	36	K.GSEEEGDITNPINLR.E	8.36E+06	9.31E+06	3.06E+06
	37	R.C(+57.02)LQSC(+57.02)QQEPDDLK.Q	1.61E+05	2.72E+05	8.44E+04

	38	K.TENPC(+57.02)AQR.C		3.26E+04	
	39	R.NTLEAAFNAEFNEIR.R	2.51E+05	0	3.58E+05
	40	R.C(+57.02)LQSC(+57.02)QQEPDDLKQK.A		5.24E+04	0
	41	K.EGALMLPH(+15.99)FNSK.A	3.91E+06	9.75E+06	5.18E+06
	42	K.G(+42.01)TGNLELVAVR.K		4.32E+04	1.87E+04
	43	R.EREEDWR.Q	2.87E+06	5.68E+05	9.49E+05
	44	K.DNVIDQIEK.Q		7.87E+05	
	45	K.GTGNLELVAVRK.E	0	2.93E+04	
	46	R.EETSRNNPFYFPSR.R			6.03E+05
	47	R.IFLAGDK.D	1.96E+05	4.12E+05	2.81E+05
	48	K.IRPEGR.E	0	6.14E+05	3.84E+05
	49	R.RVLLEENAGGEQEER.G	1.94E+05	0	1.29E+05
	50	K.L(+42.01)FEVKPDK.K		1.63E+04	
**Ara h 3**	1	R.SPDIYNPQAGSLK.T	1.64E+08	4.29E+08	1.64E+08
	2	R.IESEGGYIETWNPNNQEFEC(+57.02)AGVALSR.L	8.66E+04	2.31E+05	0
	3	K.TANDLNLLILR.W	9.54E+07	1.48E+08	7.60E+04
	4	R.VYDEELQEGH(+15.99)VLVVPQNFAVAGK.S	0	1.26E+06	
	5	R.VYDEELQEGHVLVVPQNFAVAGK.S	1.82E+07	2.02E+08	2.02E+06
	6	R.QQPEENAC(+57.02)QFQR.L	3.17E+07	5.51E+07	1.86E+07
	7	R.AHVQVVDSNGNR.V	4.84E+07	6.68E+07	1.42E+06
	8	K.SQSENFEYVAFK.T	3.44E+07	1.30E+08	1.77E+07
	9	R.RPFYSNAPQEIFIQQGR.G	1.27E+08	3.74E+08	2.51E+07
	10	K.FFVPPSQQSPR.A	2.62E+05	4.87E+06	4.18E+04
	11	R.LNAQRPDNR.I	4.74E+07	7.21E+07	2.50E+07
	12	K.T(+42.01)ANDLNLLILR.W		2.02E+05	
	13	RQQPEENAC(+57.02)QFQR.L	8.23E+05	2.74E+05	2.67E+05
	14	R.IESEGGYIETW(+15.99)NPNNQEFEC(+57.02)AGVALSR.L		3.16E+05	
	15	R.NRSPDIYNPQAGSLK.T	1.26E+05	9.88E+04	2.14E+05
	16	R.QIVQNLR.G	2.20E+06	0	1.28E+04
	17	R.S(+42.01)PDIYNPQAGSLK.T		2.13E+05	0
	18	R.V(+42.01)YDEELQEGHVLVVPQNFAVAGK.S		1.52E+05	
	19	K.NNNPFK.F	0	2.48E+06	9.01E+04
	20	R.SLPYSPYSPQSQPR.Q	6.72E+04		
	21	R.ILSPDR.K	1.34E+06	1.38E+06	5.89E+05
	22	R.L(+42.01)NAQRPDNR.I		0	5.41E+04
	23	R.Q(+42.01)QPEENAC(+57.02)QFQR.L	0	4.51E+04	
	24	R.ILSPDRK.R		5.57E+05	
**Ara h 2**	1	R.QQWELQGDR.R	9.58E+04	2.14E+05	6.47E+04
	2	R.C(+57.02)MC(+57.02)EALQQIMENQSDR.L	7.54E+04	4.13E+05	
	3	R.C(+57.02)C(+57.02)NELNEFENNQR.C	1.10E+06	1.97E+06	4.54E+05
	4	R.NLPQQC(+57.02)GLR.A	2.00E+06	3.00E+06	1.01E+06
	5	R.C(+57.02)DLEVESGGR.D	2.28E+05	3.26E+05	
	6	R.ANLRPC(+57.02)EQHLMQK.I	5.39E+05	5.82E+05	0
	7	R.C(+57.02)QSQLER.A	4.35E+05	3.86E+05	6.35E+04

	8	R.DEDSYGR.D		2.97E+04	2.17E+04
	9	R.QQEQQFK.R	6.91E+05		
	10	R.QQWELQGDRR.C	0	7.76E+04	
**Ara h 6**	1	R.VNLKPC(+57.02)EQHIMQR.I	6.98E+05	7.59E+05	2.22E+05
	2	R.C(+57.02)MC(+57.02)EALQQIMENQC(+57.02)DR.L	7.52E+04	6.45E+05	
	3	R.C(+57.02)C(+57.02)DELNEMENTQR.C	1.15E+05	2.94E+05	
	4	R.QMVQQFK.R	4.26E+05		1.86E+05
	5	R.C(+57.02)DLDVSGGR.C	1.04E+06	1.98E+06	6.95E+05
	6	R.ELMNLPQQC(+57.02)NFR.A	5.05E+05	1.44E+06	3.42E+05
